# Total RNA nonlinear polarization: towards facile early diagnosis of breast cancer†

**DOI:** 10.1039/d1ra05599b

**Published:** 2021-10-11

**Authors:** Yasser H. El-Sharkawy, Sherif Elbasuney, Sara M. Radwan, Mostafa A. Askar, Gharieb S. El-Sayyad

**Affiliations:** Head of Biomedical Engineering Department, Military Technical College Cairo Egypt; Head of Nanotechnology Research Center, Military Technical College Cairo Egypt s.elbasuney@mtc.edu.eg sherif_basuney2000@yahoo.com; Biochemistry Department, Faculty of Pharmacy, Ain Shams University Cairo Egypt; Radiation Biology Department, National Center for Radiation Research and Technology (NCRRT), Egyptian Atomic Energy Authority (EAEA) Cairo Egypt; Drug Radiation Research Department, National Center for Radiation Research and Technology (NCRRT), Egyptian Atomic Energy Authority (EAEA) Cairo Egypt Gharieb.S.Elsayyad@eaea.org.eg Gharieb.Elsayyad2017@gmail.com

## Abstract

Different cancers are caused by accumulation of numerous genetic and epigenetic changes. Recently, nonlinear polarization has been considered as a marvelous tool for several medical applications. The capability of nonlinear polarization, to monitor any changes in RNA's spectral signature due to breast cancer (BC) was evaluated. Blood samples, from healthy controls and BC patients, were collected for whole blood preparation for genomic total RNA purification. Total RNA samples were stimulated with a light-emitting diode (LED) source of 565 nm; the resonance frequency of investigated RNA samples was captured and processed *via* hyperspectral imaging. Resonance frequency signatures were processed using fast Fourier transform in an attempt to differentiate between RNA (control) and RNA (BC) *via* frequency response. RNA (BC) demonstrated a characteristic signal at 0.02 GHz, as well as a phase shift at 0.031, and 0.070 GHZ from RNA (control). These features could offer early BC diagnosis. This is the first time to describe an optical methodology based on nonlinear polarization as a facile principle to distinguish and identify RNA alterations in BC by their characteristic fingerprint spectral signature.

## Abstract 2

Nonlinear polarization has been considered as a marvelous tool for several medical applications, and the capability to monitor any changes in RNA's spectral signature due to breast cancer was evaluated by hyperspectral camera.

## Graphical abstract

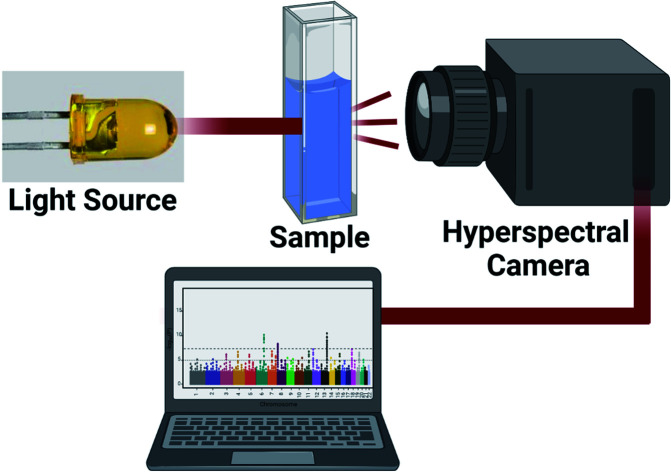

## 1. Introduction

Breast cancer (BC) is considered the leading cause of worldwide cancer deaths in females.^1^ Women with inherited BRCA1 or BRCA2 gene mutations have up to 85% risk of BC development. Mutations in several other rare susceptible genes for instance TP53, STK11, PTEN, and CHEK2 were found to contribute to induction of breast cancer.^2^ The genetic information, crucial for the identity and function of each eukaryotic cell, is stored in the DNA.^3^ The process of transcription generates RNA by using the genome as a template, depending on certain functional genomic elements.^4^ Genetic information on DNA is encoded by the four bases adenine (A), guanine (G), cytosine (C), and thymine (T).^5^ Cancer genomes are highly enriched with mutational signatures, which may be regarded as a type of base substitution (example; C: G to T: A) or as the deletion/insertion of one or more bases.^6^ Exposure to UV light can cause adjacent pyrimidines to dimerize, while oxidative damage can cause several lesions that are mutagenic if not repaired.^7^ Moreover, some of the hydrogen atoms on each of the four bases can change their location to produce a tautomer.^8^ An amino group (–NH_2_) can tautomerize to an imino form (–NH). Likewise, a keto group can tautomerize to an enol form.^5^ These mutations in BC genomes cause corresponding RNA alterations and modifications in the order of the transcribed purine and pyrimidine bases.^6,9^

Macroscopic level investigation of polarized light effects on total RNA can reveal information about structural bonds, electronic states, electronic alignments, and dielectric properties.^10^ Dielectric properties of macromolecules (as total RNA) can be determined from light absorption and emission.^11^ This means that a macromolecule exhibiting anisotropies in light absorption/emission, will exhibit characteristic polarization properties.^12^

Macromolecular polarization properties include:

❖ Depolarization: the ability to depolarize incident polarized light.^13^

❖ Di-attenuation: the dependence of light transmission on the incident polarization state.^14^

❖ Polarizance: the ability to polarize un-polarized incident light.^15^

❖ Retardance: the ability to generate a phase shift in the electric vector of incident polarized light.^16^

When the oscillating electric field is applied on macromolecules; the electric field induces dipole moment *via* partial separation of positive and negative charges.^17,18^ The macroscopic equivalence of dipole moment is the polarization *P*.^19^1
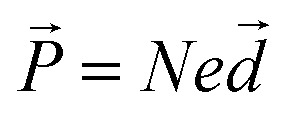
where: *N*, *e*, and *d* were number of dipoles per unit volume, electronic charge, and displacement, respectively.^20,21^

**Fig. 1:**
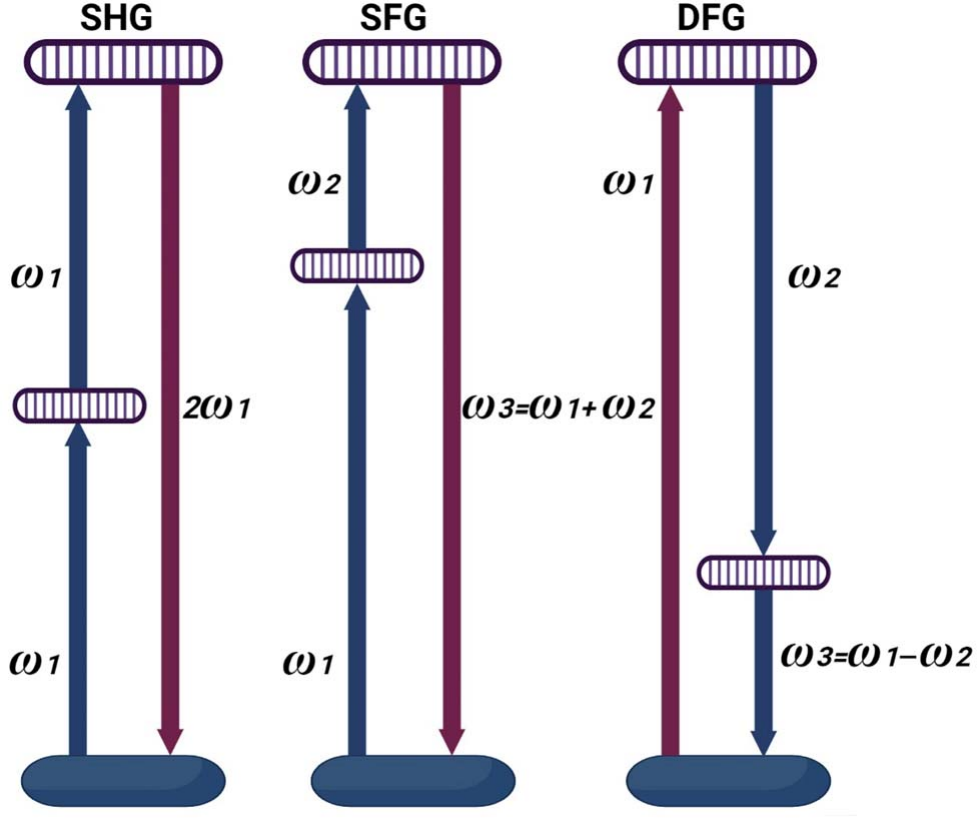
Schematic for energy level with the generation of harmonic frequencies.

The induced dipole moment can exchange energy, and can oscillate at resonant frequency with the incident electric field.^22^ The induced dipole simply re-emits electric field at the same frequency known as Rayleigh scattering.^23,24^ Linear spectroscopy cannot secure consistent information about macromolecule chemical structure (*i.e.* RNA disorder). Nonlinear spectroscopy is related to the interaction of intense light with matter.^25^ Laser sources, can secure high light intensities to modify material optical properties.^26^ Light waves can then interact with each other, exchanging momentum and energy.^27^ This interaction of light waves can result in generation of new optical frequencies.^28^ Intense irradiance can excite many molecules to the high-energy state. This yields vibrations at all frequencies corresponding to all energy differences.^29^ Nonlinear relationship between the polarization and the electric field appears only with strong fields with generation of new electric field, with new frequencies 2*ω*_1_, 2*ω*_2_, |*ω*_1_ + *ω*_2_|, and |*ω*_1_ − *ω*_2_|.^30^ The phenomenon where 2*ω*_1_ and 2*ω*_2_ frequencies are produced is called second harmonic generation (SHG).^31^ Sum frequency generation (SFG) occurred at |*ω*_1_ + *ω*_2_|. Difference frequency generation (DFG) occur at |*ω*_1_ − *ω*_2_| as shown in Fig. 1.^32^

RNA as macromolecules with an alternating electron donating and accepting groups can offer characteristic signature when stimulated with intense laser beam.^33^

Hyperspectral imaging at RNA level may provide innovative nonlinear signatures to assess RNA functions; such signature can monitor any RNA mutation due to BC.^34^ It could be considered as a way to study these molecules and answer how their structure controls the functional events inside the cell. These modalities may assist researchers in designing scenes and find out how numerous factors powerfully interact.^35^

Thus, the current study describes an optical methodology based on nonlinear polarization for spatial and spectroscopic characterization of RNA samples from BC patients and healthy volunteers to determine their spectral fingerprint signature. Latest advances in hyperspectral imaging offered unique capability for precise measurements of nonlinear polarization signature. Blood samples from five healthy controls and five BC patients were collected for whole blood preparation for total RNA purification. Total RNA samples were illuminated with LED source of 565 nm; characteristic resonance frequencies of investigated samples were captured and processed using hyperspectral camera.

## 2. Subjects and methods

### 2.1 Subjects

Five newly diagnosed BC female patients, who haven't received any chemotherapy or radiotherapy, were recruited from the clinical oncology clinic, Ain Shams University Hospitals, Cairo, Egypt. Diagnosis was based on mammogram and cell biopsy. The study also included five healthy age volunteers to serve as healthy control. Informed consent was obtained from every patient and the study was approved by the Ethical Committee of Research, Ain Shams University Hospitals. Furthermore, the study was carried out in accordance with the regulations and recommendations of the Declaration of Helsinki.

### 2.2 Laboratory analyses

For each participant, two blood samples were collected on EDTA vacutainers for whole blood preparation. Whole blood was used for complete blood count determination and total RNA purification.

#### 2.2.1 Complete blood count (CBC) analysis

Hemoglobin (Hgb), red blood cell (RBC) count, total leukocyte count (TLC) and platelet count determinations were performed using Z2TM Coulter Counter®, Analyzer, Coulter Electronics, USA.

#### 2.2.2 Total RNA purification

Total RNA was extracted and purified using a QIAamp RNA blood mini kit (Qiagen, Hilden, Germany) according to the manufacturer's protocol. Quantitation and purity assessment was performed using the NanoDrop® (ND)-1000 spectrophotometer (NanoDrop Technologies, Inc., Wilmington, DE, USA).

### 2.3 Nonlinear polarization measurements

**Fig. 2:**
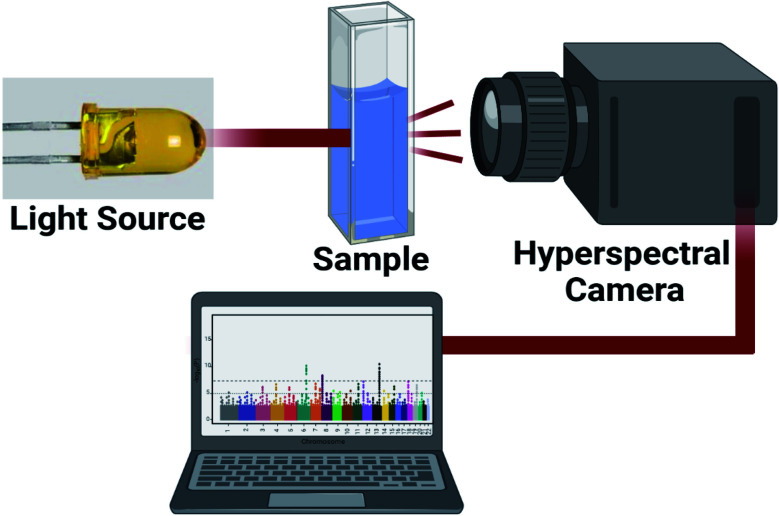
Schematic for nonlinear polarization measurements.

Nonlinear polarization can offer wealth of information about chemical and physical properties of RNA macromolecule. This approach can find attractive biomedical applications. 10 mW laser source working at 656 nm was focused using microscopic lens to illuminate investigated total RNA samples. The scattered and the re-emitted radiations were collected using hyperspectral camera (Fig. 2).

Scattered and re-emitted resonance frequency signals were processed using custom digital processing algorithm for frequency and phase domain analysis. The frequency and phase responses were calculated to discriminate RNA (control), and RNA (BC). The difference in resonance frequency signature and phase shift could be ascribed to RNA gene mutations due to BC.

### 2.4 Statistical analysis

All statistical demographic analysis was performed using SPSS version 23 software package (SPSS Inc., Chicago, IL, USA). Data are presented as mean ± standard deviation. Statistical significance between all groups was analyzed by using the ^a^*p* < 0.001, ^b^*p* < 0.01, ^c^*p* < 0.05 *vs.* RNA (BC) group. Statistical analyses graphs were drawn using Prism, version 8 (GraphPad Software, La Jolla, CA).

## 3. Results and discussions

### 3.1 Demographic data and clinical characteristics of the studied groups

The clinical and demographic data of the control and BC groups are summarized in Table 1. In agreement with previous study, the lower baseline hemoglobin and high baseline platelet level may be related to better a potential prognosis factor for breast cancer.^37^

**Table 1:** Demographic data and clinical characteristics of the studied groups^a^

Characteristics	Control group	BC group
Age (years)	51.9 ± 2.3	53.4 ± 2.5
Hemoglobin (g %)	14.1 ± 2.1^c^	13.7 ± 2.3
RBCs (×10^6^ cells per μl)	4.7 ± 0.8^c^	4.2 ± 1.1
TLC (×10^3^ cells per μl)	6.1 ± 1.9^c^	7.1 ± 1.7
Platelet count (×10^3^ cells per μl)	275 ± 29.9^b^	325 ± 37.7

a Results presented as mean ± SD.

### 3.2 Characteristic nonlinear signature

**Fig. 3:**
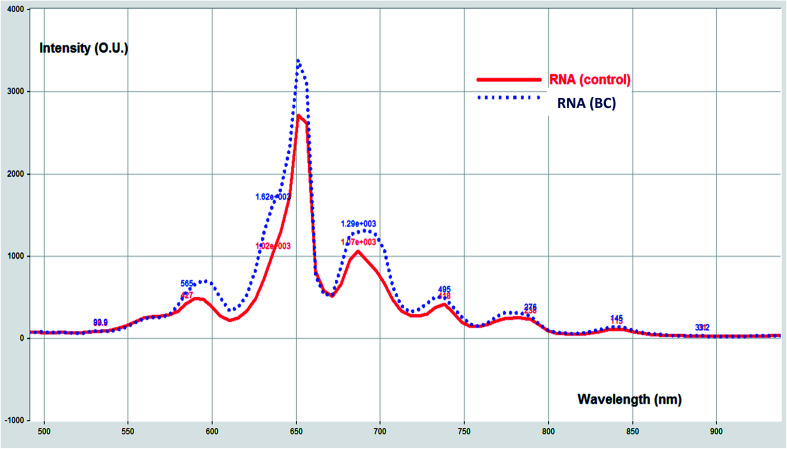
Resonance frequency (scattered and re-emitted radiations) of RNA (BC) to RNA (control) after being illuminated with laser source at 656 nm.

The change in harmonic oscillation response could be correlated to chemical change and disorder of absorber size, location, and organization.^36^ This signature could provide information about RNA mutation (chemical structure changes) related to BC. This approach could offer early diagnosis of BC as well as early treatment. Nonlinear resonance frequency response of investigated RNA samples, including RNA (control) and RNA (BC) are represented in ESI (Fig. S1, S2, and S3).† The averaged signal of investigated five health control and five BC patients are demonstrated in Fig. 3.

**Fig. 4:**
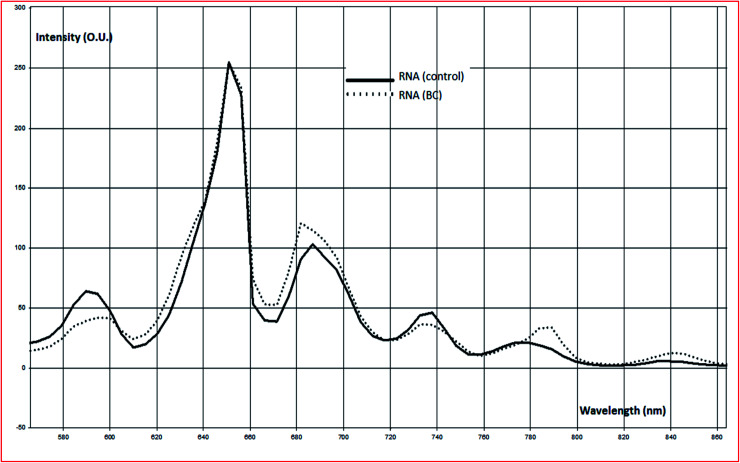
Normalized resonance frequencies (scattered and re-emitted radiations) of RNA (BC) to RNA (control) after being illuminated with laser source at 656 nm.

It is obvious that each macromolecule demonstrated its characteristic nonpolarized resonance frequency signature. Resonance RNA (BC) frequency signature differ slightly from RNA (control). In an attempt to determine the spectral oscillation signature for investigated RNA samples; normalized resonance frequency signatures were calculated to eliminate the effect of polarized light intensity (Fig. 4).

Due to the changes in molecular electronic configuration, absorber size in the order (nm); the re-emitted signature will be in the range of visible and near IR.

### 3.3 Non-polarized resonance frequency signature

RNA alterations are accompanied with changes in its optical and dielectric properties when stimulated with laser light. Resonance frequency radiation could offer a fingerprint signature of the macromolecule chemical structure and physical properties. Table 2 demonstrates the harmonic resonance frequency for RNA (BC) and RNA (control).

**Table 2:** Characteristic light polarization signature of investigated RNA samples

Sample	Rayleigh scattering (nm)	Re-emitted radiation (2^nd^ harmonic) (nm)	Re-emitted radiation (3^rd^ harmonic) (nm)	Re-emitted radiation (4^th^ harmonic) (nm)	Re-emitted radiation (5^th^ harmonic) (nm)
RNA (control)	656	692.1	738.0	778.9	840.2
RNA (BC)	656	692.0	738.0	778.9	840.2

Chemical structural arrangements that create differential absorption of different polarization states can exhibit light attenuation. The resonance frequency attenuation of investigated RNA samples is demonstrated in Table 3.

**Table 3:** Attenuation of transmitted light polarization (scattered and re-emitted) of investigated samples

Sample	Rayleigh scattering (intensity a.u.)	Re-emitted radiation (2^nd^ harmonic) (intensity)	Re-emitted radiation (3^rd^ harmonic) (intensity)	Re-emitted radiation (4^th^ harmonic) (intensity)	Re-emitted radiation (5^th^ harmonic) (intensity)
RNA (control)	3472.7	1208.4	417.9	841.0	85.0
RNA (BC)	3915.2^c^	2526.7^b^	1244.4^a^	604.3^c^	166.9^a^

### 3.4 Frequency changes and phase differences

**Fig. 5:**
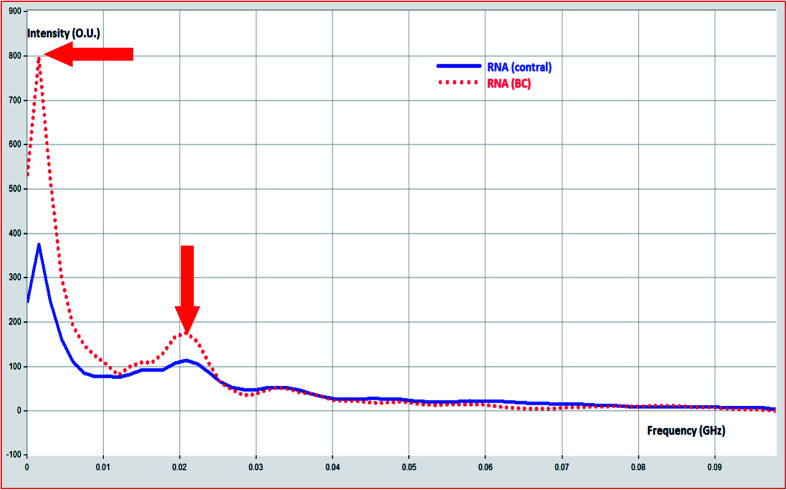
Determination of frequency change due to change in RNA structure in BC.

The scattered and re-emitted radiation of total RNA samples were processed using fast Fourier transform in an attempt to differentiate between RNA (control) and RNA (BC) *via* frequency response change (Fig. 5).

**Fig. 6:**
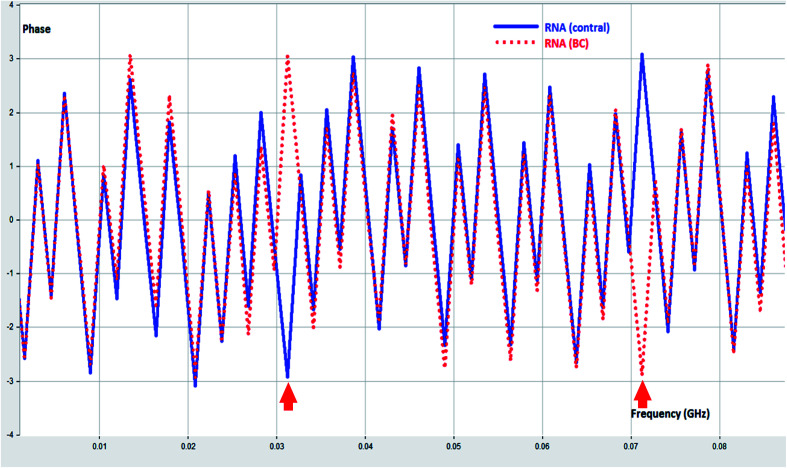
Phase difference between RNA (control) and RNA (BC).

RNA (BC) demonstrated characteristic frequency signal from RNA (control). RNA (BC) demonstrated frequency band 0.004–0.01 GHz, and characteristic signal at 0.02 GHz. The phase change of RNA (BC) to RNA (control) was calculated from frequency signal as demonstrated in Fig. 6.

RNA (BC) demonstrated phase shift at 0.031, and 0.07 GHZ. These phase shift could be ascribed to change in chemical structure and dielectric properties due to BC.

**Fig. 7:**
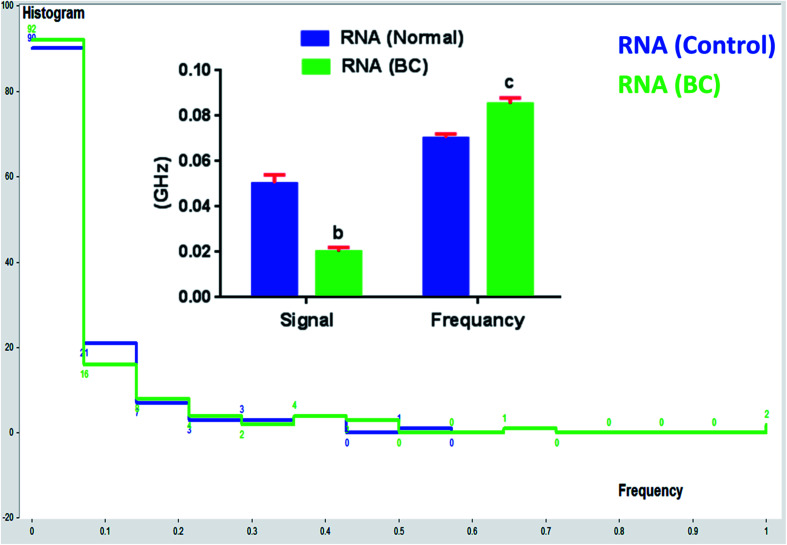
The calculated histogram analysis for RNA (control), and RNA (BC).

Statistical analysis of obtained results was performed; the histogram for the frequency distribution of 5 signal observations of RNA (control), and RNA (BC) response was calculated. The mean values for the RNA (control), and RNA (BC) are 0.0783 and 0.0852, respectively. The standard deviation for the RNA (control) and RNA (BC) are 0.0020 and 0.0027, respectively (Fig. 7). It can be concluded that reproducible results were obtained for all investigated samples.

## 4. Conclusion

BC included RNA mutations with changes in RNA chemical structure, and physical properties (polarizability, and total refractive index). This is the first time to report an optical approach based on nonlinear polarization to identify RNA alterations in BC *via* their specific fingerprint nonlinear resonance frequency signature. RNA (BC) demonstrated characteristic frequency signal and phase shift from RNA (control). RNA (BC) demonstrated characteristic signal at 0.02 GHz; furthermore RNA (BC) demonstrated phase shift at 0.031, and 0.07 GHZ from RNA (control). We hope that our study will assist more researchers and pave the way for finding out how numerous factors powerfully interact in order to utilizing non-linear polarization in direct, fast, non-invasive diagnosis of BC.

## Ethics approval

All experimental and investigation trials was approved and validated from “Ain Shams University” – Medical College – Ethics Committee.

## Conflicts of interest

The authors stated and declare that there is no conflict or competing of interests.

## Supplementary Material
